# Parental exercise is associated with Australian children's extracurricular sports participation and cardiorespiratory fitness: A cross-sectional study

**DOI:** 10.1186/1479-5868-2-3

**Published:** 2005-04-06

**Authors:** Verity Cleland, Alison Venn, Jayne Fryer, Terence Dwyer, Leigh Blizzard

**Affiliations:** 1 Menzies Research Institute, University of Tasmania, Hobart, Australia

## Abstract

**Background:**

The relationship between parental physical activity and children's physical activity and cardiorespiratory fitness has not been well studied in the Australian context. Given the increasing focus on physical activity and childhood obesity, it is important to understand correlates of children's physical activity. This study aimed to investigate whether parental exercise was associated with children's extracurricular sports participation and cardiorespiratory fitness.

**Methods:**

The data were drawn from a nationally representative sample (n = 8,484) of 7–15 year old Australian schoolchildren, surveyed as part of the Australian Schools Health and Fitness Survey in 1985. A subset of 5,929 children aged 9–15 years reported their participation in extracurricular sports and their parents' exercise. Cardiorespiratory fitness was measured using the 1.6 km (1-mile) run/walk and in addition for children aged 9, 12 or 15 years, using a physical work capacity test (PWC_170_).

**Results:**

While the magnitude of the differences were small, parental exercise was positively associated with children's extracurricular sports participation (*p *< 0.001), 1.6 km run/walk time (*p *< 0.001) and, in girls only, PWC_170 _(*p *= 0.013). In most instances, when only one parent was active, the sex of that parent was not an independent predictor of the child's extracurricular sports participation and cardiorespiratory fitness.

**Conclusion:**

Parental exercise may influence their children's participation in extracurricular sports and their cardiorespiratory fitness levels. Understanding the correlates of children's extracurricular sport participation is important for the targeting of health promotion and public health interventions, and may influence children's future health status.

## Background

An important component of children's total physical activity comes from extracurricular sports participation. Extracurricular sports are those organised and non-organised sports played outside of the school curriculum, and form an element of children's discretionary physical activity. Sports participation in childhood and adolescence is important because it is thought to be associated with lower levels of antisocial behaviour [1,2], higher levels of positive emotional wellbeing [3] and greater participation in sport in adulthood [4].

Extracurricular sports participation has substantially decreased in Australian children over the last 15 years. In 1985, 91% of boys and 90% of girls participated in at least one extracurricular sport [5], whereas in 2000 this had decreased to 71% of boys and 58% of girls participating in at least one extracurricular sport [5,6]. Over the same period, the prevalence of childhood overweight and obesity has increased, with Australian children in 1995 being nearly twice as likely to be overweight and more than three times as likely to be obese as children in 1985 [7].

With the increasing rates of childhood overweight and obesity [7], and suspected declines in levels of overall physical activity, it is important to explore correlates of children's physical activity. A recent review of these correlates recommended more research examining the inconsistent associations seen in previous studies between children's physical activity and parental physical activity [8]. There have also been inconsistent findings in studies that have assessed whether the sex of the physically active parent is associated with children's physical activity [9-12]. These inconsistencies are possibly due to methodological differences, which make it difficult to compare results directly. For instance, studies that have used accelerometers to objectively measure physical activity in children and their parents have found significant associations between children's and parental physical activity [9,13,14], as have studies that have used children's reports of their parent's physical activity [10,15,16]. Studies that have used self-reported parental physical activity have generally found no associations[11,17-20]. A number of these studies, however, had limited sample sizes [9,13,14,17], reducing the statistical power and limiting the ability to draw firm conclusions.

Few studies have explored the relationship between parental exercise and children's extracurricular sports participation and cardiorespiratory fitness levels. In the two studies that have explored this relationship [11,18], no association was found between parental physical activity and children's physical fitness, as measured by maximal oxygen uptake [11] and 1-mile run time [18]. However, it may be expected that parental exercise plays a role in children's physical fitness through a variety of mechanisms, including role modeling and genetic associations. No research to date has used a population-based sample, nor has this relationship been assessed in the Australian setting, which may have unique sociocultural features.

This study aimed to examine the relationship between children's extracurricular sports participation, cardiorespiratory fitness and parental exercise in a large, nationally representative sample of Australian children aged 9–15 years. We hypothesise that children who report having more active parents are involved in a greater number of extracurricular sports and have higher levels of cardiorespiratory fitness than children who report having less active parents. We also hypothesise that where only one parent is reported as active, that the sex of that parent influences children's extracurricular sport participation and cardiorespiratory fitness.

The Australian Council for Health and Physical Education Research's Australian Schools Health and Fitness Survey (ASHFS), conducted in 1985, remains the largest population-based study of children in Australia with extensive health and fitness measures and a high response rate. Due to this high response rate and population-based approach, results from this study are less likely to be subject to selection bias. Data from this study provide information on children and adolescents from a wide age range (9–15 years) and provide the opportunity to compare with current and future trends.

## Methods

### Participants

In 1985, a nationally representative sample of 8,484 children aged 7–15 years participated in the ASHFS, which gathered extensive measures of health and fitness through various field and technical tests, questionnaires and blood samples. The sampling design and sampling techniques are described in detail elsewhere [21]. Briefly, the first stage of sampling involved selecting schools with probability proportional to enrolment, and the second stage involved simple random sampling within each age/sex category to select children from within those schools. This sampling plan was designed to yield a self-weighted sample. Ninety per cent of schools approached agreed to participate (n = 109), and informed consent was obtained from the parents of 77.5% of the students selected in the initial sample. Participants aged 9–15 years (n = 6,659) were surveyed about their extracurricular sports participation and their parents' exercise. Children aged 7 and 8 years were not included in this aspect of the survey, because they were considered too young to reliably complete the questionnaire. Trained data collectors read questionnaire instructions to children in groups of four and supervised the completion of the questionnaire. Approval was granted to contact schools by the State Directors General of Education, and parental and child consent was required for inclusion in the study.

### Predictor Variable

#### Children's report of parental exercise

In the student questionnaire, children were asked to report their parents' involvement in exercise. Specifically, children were asked, "Does your father exercise regularly (2 or more times a week)?" and "Does your mother exercise regularly (2 or more times a week)?". The examples of jogging, playing sport, doing exercises, going to a gym and doing aerobics were provided. Children's responses were classified as 'both parents active', 'mother active only', 'father active only' and 'both parents inactive'. Children who responded 'yes' for both their mother and their father were categorised as 'both parents active' (n = 1,183). If the child responded 'yes' for mother but 'no', 'don't know' or had missing data for their father, they were categorised as 'mother active only' (n = 1,055); 'father active only' was defined analogously (n = 1,192). If the child reported 'no' for both parents, or 'no' for one parent and 'don't know' or had missing data for the other parent, this resulted in the category 'both parents inactive' (n = 2,499). Children who responded 'don't know' or had missing data for both parents were categorised as 'incomplete' and excluded from the analyses (n = 485).

### Outcome Variables

#### Children's extracurricular sports participation

The children were asked to list all the sports they had played regularly in the previous year, including sports played with an organised team, group, club or school, but not including sports played in physical education class at school. Children were provided with the opportunity to list up to six sports.

#### Cardiorespiratory fitness

Two measures of children's cardiorespiratory fitness were used. All children completed a timed 1.6 km (1 mile) run/walk. Children aged 9, 12 or 15 years also participated in a physical work capacity (PWC_170_) test on a Monark bicycle ergometer as a continuous test (n = 2,619). The workload corresponding to a heart rate of 170 beats per minute was predicted by linear regression from the heart rates recorded at the three submaximal loads [22]. This score was then divided by the child's weight in kilograms to take body size into consideration.

### Potential Confounders

#### Area-Level Socioeconomic status

Numerous studies have found that indicators of socioeconomic status (SES), such as education, occupation and income, are associated with adult physical activity levels [23], although findings are not so clear in children [8,24]. Area-level socioeconomic status (SES) was therefore estimated in this study based on the Australian Bureau of Statistics (ABS) socioeconomic index for areas (SEIFA). The SEIFA is a summary of five indices designed to measure different aspects of SES by geographical area, based on questions asked in the Australian population census [25]. The ABS classified all Australian postcodes into one of four categories (low, medium-low, medium-high, high) based on one of these indices, the index of relative socioeconomic disadvantage (IRSD). The IRSD is constructed from items in the population census, including income, educational attainment, employment and occupation type. Using the 1981 census data, approximately 25% of Australia's population falls into each of the four IRSD categories. In this analysis, the IRSD was assigned to the child's residential postcode. In some cases, information on the child's residential postcode was unavailable and these children were therefore excluded from the analyses (n = 115).

#### School type

Children attended government (public or non-fee paying), independent (private or fee-paying) or Catholic (private or fee-paying) schools. The type of school attended was considered because educational policies and ethos may vary between school sectors, which may influence children's participation in extracurricular sport.

### Analysis

Two-sample t-tests were used to compare the sample characteristics of boys and girls. Linear regression methods were used to determine whether parental exercise was associated with children's extracurricular sports participation and cardiorespiratory fitness (1.6 km run & PWC_170_). To adjust for the effects of clustering of children within schools, the estimates of standard errors were computed using the Taylor-series approximation. For these analyses, three indicator (dummy) variables were included for parental exercise (both parents active, mother active only, father active only), with the reference group being 'both parents inactive'. P-values corresponding to the test of association (multiple-partial F-test) are reported. The analyses were restricted to the 5,929 children who provided complete information on extracurricular sports participation (representing 96% of those who completed the questionnaire), and the number of sports reported was treated as a continuous variable.

Similar methods were used to examine the relationship between children's extracurricular sports participation and each of the cardiorespiratory fitness measures. As a test for trend, we categorised the number of sports into four levels and report the test of significance of a single predictor with values ranging from one (at most one sport) to four (four or more sports).

To assess whether associations between parental exercise and children's extracurricular sports participation differed by the sex of the active parent, 'mother active only' was used as the reference category and compared to the category 'father active only'. Additionally, 'both parents active' was compared to 'both parents inactive'. These analyses are presented in the text.

All analyses were stratified by sex to assess whether associations varied between boys and girls. All analyses were routinely adjusted for age, SES and school type because these variables confounded associations in a number of the analyses. We tested for effect modification by age by including a (child's age × parents' exercise) product term in each final model, and found evidence of it only in one isolated instance (the model relating girls' participation in extracurricular sport to mothers' exercise, data not reported). Stata software (Version 8.2) was used for all analyses (28).

## Results

### Sample characteristics

The number and percentage of boys and girls in each age group, school type, SES group, proportion overweight and obese, and parental exercise category are presented in Table 1 (n = 5,929). The majority of children attended government schools, one fifth attended Catholic schools and the remaining children attended independent schools. 10.9% of boys and 12.0% of girls were considered overweight or obese. Boys and girls reported parents' exercise similarly, with 43.4% of boys and 40.9% of girls reporting that they had two physically inactive parents.

**Table 1:** Total number and percentage of children participating in the ASHFS by age, school type, socioeconomic status (SES), weight status, parental exercise and number of sports played

	**Boys **(N = 3,256)		**Girls **(N = 3,158)	
	n	%	n	%

**Age**				
9	476	14.6	482	15.3
10	479	14.7	489	15.5
11	473	14.5	475	15.0
12	473	14.5	479	15.2
13	454	13.9	426	13.5
14	453	13.9	400	12.7
15	448	13.8	407	12.9
**School Type**				
Government	2,403	73.8	2,335	73.9
Catholic	655	20.1	677	21.4
Independent	198	6.1	146	4.6
**SES**				
Low	310	9.7	272	8.8
Medium-low	1,230	38.5	1,197	38.6
Medium-high	914	28.6	886	28.6
High	742	23.2	748	24.1
Weight status*				
Under/acceptable weight	2,899	89.0	2,778	88.0
Overweight	301	9.2	335	10.6
Obese	56	1.7	45	1.4
**Parental Exercise**				
Both parents active	553	18.4	630	21.5
Mother active only	518	17.3	537	18.3
Father active only	627	20.9	565	19.3
Both parents inactive	1,303	43.4	1,196	40.9
Incomplete data	255	7.8	230	7.3
**Number of sports**				
0 sport	184	5.7	230	7.3
1 sport	586	18.0	691	21.9
2 sports	881	27.1	857	27.1
3 sports	740	22.7	658	20.8
4 sports	484	14.9	404	12.8
5 sports	235	7.2	187	5.9
6 sports	146	4.5	131	4.2

* Cut-off points for BMI in childhood based on international data linked to the widely accepted adult cut-off points for overweight (BMI 25.0–29.9 kg/m^2^) and obesity (≥ 30.0 kg/m^2^) [42]

The mean (standard deviation) weight, height, number of sports played, time taken to complete a 1.6 km run/walk and PWC_170 _(kgm.kg-1.min-1) are presented in Table 2. Compared to girls, boys in this sample were heavier (*p *= 0.008), taller (*p *< 0.001), played more sport (*p *< 0.001), completed the 1.6 km run/walk in less time (*p *< 0.001), and had higher PWC_170 _scores (*p *< 0.001).

**Table 2:** Weight, height, number of extracurricular sports played, time to complete a 1.6 km run/walk, and PWC_170 _(kgm.kg^-1^.min^-1^) of children aged 9–15 years in the ASHFS 1985

Mean (standard deviation)	Boys	Girls
Weight *(kg)*	44.2 (12.8)	43.4 (11.4)
Height *(m)*	152.3 (13.9)	150.3 (11.6)
Extracurricular sports* *(n)*	2.6 (1.5)	2.4 (1.5)
1.6 km run/walk time *(mins & secs)*	8 m 6 s (1 m 30 s)	9 m 48 s (1 m 42 s)
*PWC*_*170 *_*(kgm.kg^-1^.min^-1^)*	14.7 (3.5)	11.2 (3.0)

*Number of extracurricular sports played

### Parental exercise and children's extracurricular sports participation

Table 3 shows that parental exercise was associated with children's extracurricular sports participation in both boys (*p *< 0.001) and girls (*p *< 0.001). Children who had two active parents participated in, on average, 0.6 more sports than those with two inactive parents. Where only one parent was active, the number of sports boys and girls participated in was significantly higher than where neither parent was active (*p *< 0.001 for all associations). Interestingly, the sex of that parent made no significant difference to the number of extracurricular sports played by either boys (*p *= 0.66) or girls (*p *= 0.21).

**Table 3:** Number of extracurricular sports played by children by parental exercise

Parental exercise	Boys	Girls
Both active	3.0 (0.07)	2.8 (0.08)
Mother active only	2.7 (0.07)	2.5 (0.09)
Father active only	2.7 (0.07)	2.6 (0.08)
Both inactive	2.4 (0.06)	2.2 (0.06)
Test of association	*p *< 0.001	*p *< 0.001

Values are least squares means (standard error) adjusted for age, SES & school type

### Parental exercise and children's 1.6 km run/walk time

Table 4 shows the association between parental exercise and the number of minutes taken by boys and girls to complete a 1.6 km run/walk. On average, boys who had two physically active parents completed the 1.6 km run/walk 18 seconds faster than boys of two physically inactive parents (*p *< 0.001), and girls of two physically active parents completed the 1.6 km run/walk on average 24 seconds faster than girls of two physically inactive parents (*p *< 0.001). Compared to children with two inactive parents, girls whose mother only was active, and boys whose father only was active, completed the 1.6 km run/walk faster (*p *= 0.05 and *p *= 0.08 respectively). There was no significant difference in the 1.6 km run/walk time between children with no active parents compared to boys whose mother only was active (*p *= 0.41) and girls whose father only was active (*p *= 0.10). When only one parent was active, the sex of that parent made no significant difference to the time taken to complete the 1.6 km run/walk by boys (*p *= 0.53) and girls (*p *= 0.85).

**Table 4:** Average minutes and seconds (standard deviation) taken by children to complete a 1.6 km run/walk by parental exercise

Parental exercise	Boys	Girls
Both active	7 m 48 s (4.2 s)	9 m 30 s (4.8 s)
Mother active only	8 m 6 s (4.2 s)	9 m 42 s (5.4 s)
Father active only	8 m 0 s (3.6 s)	9 m 48 s (6.0 s)
Both inactive	8 m 6 s (3.0 s)	9 m 54 s (5.4 s)
Test of association	*p *< 0.001	*p *< 0.001

Values are least squares means (standard error) adjusted for age, SES & school type

### Parental exercise and children's PWC_170_

A subset of the sample, which included those children aged 9, 12 and 15 years, participated in a PWC_170 _test. Table 5 shows that parental exercise was significantly associated with girls' PWC_170 _(*p *= 0.002), but not with boys' (*p *= 0.55). Girls who had two physically active parents had PWC_170 _scores on average 0.7 kgm.kg^-1^.min^-1 ^greater than girls of two physically inactive parents (*p *= 0.005). When only one parent was active, the sex of that parent did make a difference to PWC_170_. Girls achieved significantly higher scores if their mother only was active than if their father only was active (*p *= 0.02). While having an active mother only was associated with higher PWC_170 _scores in boys than having an active father only, this difference was not statistically significant (*p *= 0.63). Similarly, while girls with an active mother had higher scores than girls with two active parents, this difference was not statistically significant (*p *= 0.56).

**Table 5:** PWC_170 _(kgm.kg^-1^.min^-1^) in children by parental exercise

Parental exercise	Boys	Girls
Both active	14.9 (0.25)	11.6 (0.21)
Mother active only	14.7 (0.28)	11.7 (0.24)
Father active only	14.5 (0.22)	11.0 (0.27)
Both inactive	14.7 (0.23)	10.9 (0.18)
Test of association	*p *= 0.55	*p *= 0.002

Values are least squares means (standard error) adjusted for age, SES & school type

### Children's extracurricular sports participation and cardiorespiratory fitness

In general, participation in a greater number of extracurricular sports was reflected by a higher level of cardiorespiratory fitness. Table 6 shows the average minutes taken to complete a 1.6 km run/walk and the PWC_170 _score according to the number of extracurricular sports played by children. A dose-response relationship existed between extracurricular sports participation and minutes taken to complete a 1.6 km run/walk in all children (*p *< 0.001), between extracurricular sports participation and PWC_170 _in girls (*p *< 0.001), and a borderline association between extracurricular sport participation and PWC_170 _in boys (*p *= 0.09). Boys who played four or more sports completed the 1.6 km run/walk on average 30 seconds faster than boys who played at the most one sport, and girls who played four or more sports completed the 1.6 km run/walk on average 42 seconds faster than girls who played at the most one sport. Boys who played four or more sports had PWC_170 _scores on average 0.4 kgm.kg^-1^.min^-1 ^greater than boys who played at the most one sport, and girls who played four or more sports had PWC_170 _scores on average 1.3 kgm.kg^-1^.min^-1 ^greater than girls who played at most one sport.

**Table 6:** Children's extracurricular sports participation by cardiorespiratory fitness, measured by a 1.6 km run/walk time (minutes) and PWC170 (kgm.kg^-1^.min^-1^)

Number of sports	Cardiorespiratory Fitness			
	
	1.6 km run/walk (mins and secs)		PWC170 (kgm.kg-1.min-1)	
	
	Boys	Girls	Boys	Girls
0–1 sport	8 m 18 s (3.6 s) (n = 727)	10 m 6 s (4.8) (n = 838)	14.5 (0.21) (n = 339)	10.6 (0.17) (n = 382)
2 sports	8 m 12 s (3.0 s) (n = 837)	9 m 54 s (4.2) (n = 788)	14.6 (0.17) (n = 358)	11.0 (0.15) (n = 358)
3 sports	8 m 0 s (3.0 s) (n = 696)	9 m 42 s (4. 2) (n = 607)	14.7 (0.16) (n = 314)	11.5 (0.16) (n = 268)
4+ sports	7 m 48 s (3.6 s) (n = 814)	9 m 24 s (4.8) (n = 652)	14.9 (0.19) (n = 322)	11.9 (0.21) (n = 278)
Test for trend	*p *< 0.001	*p *< 0.001	*p *= 0.09	*p *< 0.001

Values are least squares means (standard error) adjusted for age, SES & school type

## Discussion

The findings from this study suggest that parental exercise is positively associated with children's extracurricular sports participation and cardiorespiratory fitness. Having two physically active parents was associated with participation in a significantly higher number of extracurricular sports and with significantly greater cardiorespiratory fitness, compared to having two physically inactive parents. When only one parent was active, the sex of that parent was not an independent predictor of the child's extracurricular sports participation and cardiorespiratory fitness in most instances. These associations remained after adjusting for age, SES and school type.

While the magnitude of the differences observed in the current study were small, they may still be of public health significance. In adults, clear associations have been demonstrated between physical activity and cardiorespiratory fitness and various health outcomes, including all-cause mortality [26,27] and cardiovascular disease [28-30]. Other research has suggested that physical activity behaviours in childhood may predict physical activity behaviours in adulthood [4,31,32]. The contribution that childhood behaviours make to adult health is unclear, but it is possible that small associations such as those seen in the present study may play a role.

There are a number of possible mechanisms, also identified in other studies, through which parental exercise may influence children's extracurricular sports participation. Firstly, it is plausible that parents act as role models for children's extracurricular sports participation. When children observe that their parents are actively involved in and value sport and exercise, they may adopt these values and behaviours themselves. Secondly, a recent review of correlates of children and adolescent's physical activity found parental encouragement and support to be more strongly associated with children and adolescents' participation in physical activity than parental role modeling [8]. It is possible that physically active parents provide more support and encouragement for their children to be active and influence their children's activity levels in this way. Thirdly, it may be that a genetic predisposition to physical activity exists. In a recent review, Beunen and Thomis reported that the heritability coefficients for sports participation ranged from 0.35–0.83, and that children who had a parent active in sport had 1.2–5.8 times the odds of participating in sport than children whose parents were not active in sport [33].

In the current study, an association was also seen between parental exercise and children's cardiorespiratory fitness. It is possible that by influencing children's physical activity, parental exercise may indirectly influence children's fitness. However, a recent analysis found that while more active children tended to be fitter, children's physical activity only had a modest correlation with fitness (*r *= 0.17) [34]. It is also possible that parental fitness may influence children's fitness through genetic mechanisms. Results from studies assessing familial resemblance of cardiorespiratory fitness have found varying results, with the contribution of genetic factors ranging from 20–50% [35,36].

Interestingly, our findings are consistent with the results of other studies that have used similar measures of physical activity. For instance, both Anderssen and Wold [15] and Gregson and Colley [10] measured children's physical activity using self-reported questionnaires and measured parental physical activity using a questionnaire with children. Our findings also parallel the results of studies that have used accelerometers to objectively measure physical activity in children and adults [9,13]. However, studies that have used a parental self-report of physical activity have generally found no association between children's physical activity and parental physical activity [11,16-18,20].

Little research has explored the reliability and validity of children's reports of their parents' physical activity. One study of 198 Grade 7–9 students found that children's report of their parents' physical activity correlated with parental self-reported physical activity at low to moderate levels (r = 0.16–0.47), except in Grade 9 girls (r = -0.01) [16]. Another study of 755 Norwegian families demonstrated a moderate correlation between parents' self-reports of physical activity and their 13 year-old children's reports of parental physical activity (*r *0.42–0.54, *p *< 0.001) [37]. This suggests that the children in the current study were likely to report reasonable estimates of their parents' exercise. It is possible that children's perceptions of their parents' exercise behaviours may have a greater influence on children's physical activity than parents' exercise behaviours themselves. However, studies that have assessed parental physical activity objectively have found similar associations as the current study.

The current study found very little difference between the association of mothers' exercise compared to fathers' exercise with children's extracurricular sports participation and cardiorespiratory fitness, with the exception of a stronger association of mothers' exercise and girls' PWC_170_. These findings generally suggest that as long as one parent is active, the sex of the active parent makes little difference to extracurricular sports participation and cardiorespiratory fitness, with the exception of PWC_170 _in girls, where having an active mother only was associated with greater fitness than having an active father only.

These results contrast with previous research that found fathers' activity to be more influential than mothers' activity [9,12], mothers' activity to be more influential on girls' physical activity [10,11], mothers' activity to be more influential than fathers' activity on boys' physical activity [38] and parents of the same sex as the child to have the most influence on their child's physical activity [39]. These inconsistencies may be due to different definitions and assessments of children's physical activity. In the current study, the extracurricular sports component of physical activity was the main outcome factor, whereas previous studies have examined children's leisure time physical activity. Previous research has found that parents play a strong 'gate keeping' role in their children's extracurricular sports participation [18]. Parents are the usual providers of transport, equipment and funding typically not required for general and school-based physical activity. It is possible that as long as one parent provides support for extracurricular activity, the sex of that parent is not important. The sex of the active parent may be more important in influencing other domains of children's physical activity, such as active play or household activity. The differences in findings between this and other studies may also be related to the differing measures of physical activity. Some measures may "capture" some activities – such as high intensity, structured activities – better than others, and it is plausible that men and women participate in different types of activities that are "captured" differently.

A limitation of this study was that regular parental exercise was defined in the ASHFS 1985 survey as two or more times per week, while guidelines dating back to the 1960s recommend physical activity on three to five days per week for health benefits [40]. Although the ASHFS definition of regular physical activity may not meet current guidelines, this data still provides an estimate of those parents who participated in regular exercise and those who did not. A second limitation is that this study did not collect information on the frequency, duration or intensity of the extracurricular sports. It is possible that children who listed six sports may only participate in these occasionally, whereas children who listed one sport may participate every day for a number of hours. Additionally, children were asked to lists sports they had played "regularly" in the past year, yet a definition of "regularly" was not provided. The interpretation of this question by the children may therefore be variable. However, it was interesting to note the strong and consistent relationship seen between the number of extracurricular sports listed and both cardiorespiratory fitness measures in boys and in girls.

As evidenced in the current study, parental exercise may be important for children's extracurricular sports participation and cardiorespiratory fitness, which may influence children's health later in life. In the nationwide Active Australia survey, parents were deemed one of six population groups at risk of physical inactivity [41]. Those parents with one or more children under the age of 18 living at home were 20% less likely to be considered sufficiently active than those adults without children [41]. The low national levels of parental physical activity in Australia are consequently of concern. While these data are historical, we believe it would be useful for other researchers to use the data reported here to assess whether cultural changes, technological advances and increases in overweight and obesity have impacted on the apparent association between children's extracurricular sports participation, cardiorespiratory fitness and parental exercise.

## Conclusion

The results of this cross-sectional study suggest that parental exercise may influence children's participation in extracurricular sport and their cardiorespiratory fitness. While the magnitude of the differences seen was small, these differences may be important for future health. Because Australian parents are also a group at risk of physical inactivity, targeting parents may provide health benefits for the whole family.

## Competing interests

The author(s) declare that they have no competing interests.

## Authors' contributions

VC conceptualised and drafted the paper. AV contributed to the conceptualisation of the paper, interpretation of the data and critical revision of the paper. JF provided statistical support and critical revision of the paper. TD was involved in the design, management and data collection in the ASHFS, contributed to the interpretation of the data and provided critical revision of the paper. LB provided statistical guidance and critical revision of the paper. All authors read and approved the final manuscript.
